# Human Infections with Non-O157 Shiga Toxin–producing *Escherichia coli*, Switzerland, 2000–2009

**DOI:** 10.3201/eid1702.100909

**Published:** 2011-02

**Authors:** Ursula Käppeli, Herbert Hächler, Nicole Giezendanner, Lothar Beutin, Roger Stephan

**Affiliations:** Author affiliations: University of Zurich, Zurich, Switzerland (U. Käppeli, H. Hächler, N. Giezendanner, R. Stephan);; Federal Institute for Risk Assessment, Berlin, Germany (L. Beutin)

**Keywords:** Shiga toxin, Escherichia coli, bacteria, human infections, non-O157, STEC, Switzerland, research

## Abstract

We characterized 97 non-O157 Shiga toxin (stx)–producing *Escherichia coli* strains isolated from human patients during 2000–2009 from the national reference laboratory in Switzerland. These strains belonged to 40 O:H serotypes; 4 serotypes (O26:H11/H^–^, O103:H2, O121:H19, and O145:H28/H^–^) accounted for 46.4% of the strains. Nonbloody diarrhea was reported by 23.2% of the patients, bloody diarrhea by 56.8%. Hemolytic uremic syndrome developed in 40.0% of patients; serotype O26:H11/H^–^ was most often associated with this syndrome. Forty-five (46.4%) strains carried *stx*2 genes only, 36 strains (37.1%) carried *stx*1, and 16 (16.5%) strains carried *stx*1 and *stx*2. Genes encoding enterohemolysin and intimin were detected in 75.3% and 70.1% of the strains, respectively. Resistance to >1 antimicrobial agent was present in 25 isolates. High genetic diversity within strains indicates that non-O157 stx–producing *E. coli* infections in Switzerland most often occurred as single cases.

Shiga toxin (stx)–producing *Escherichia coli* (STEC) is among the most common causes of food-borne diseases (*1*). This organism is responsible for several human gastrointestinal illnesses, including nonbloody or bloody diarrhea. Especially in children, these diseases may be affected by neurologic and renal complications, including hemolytic uremic syndrome (HUS). Most outbreaks and sporadic cases of bloody diarrhea and HUS have been attributed to strains of STEC serotype O157:H7. However, in Europe and recently in the United States, the role of non-O157 STEC strains (e.g., O26:H11/H^–^, O91:H21/H^–^, O103:H2, O111:H^–^, O113:H21, O121:H19, O128:H2/H^–^, and O145:H28/H^–^) as causes of HUS, bloody diarrhea, and other gastrointestinal illnesses is being increasingly recognized (*1*).

The common feature and main virulence factor of STEC is production of stx1 or stx2 proteins. Human virulent STEC strains often may also contain other virulence factors such as intimin (*eae*), a protein essential for the intimate attachment and the formation of attaching and effacing lesions on gastrointestinal epithelial cells, and *E. coli* hemolysin (*ehxA*) (*2*).

Little data are available for clinical non-O157 STEC infections in humans, including those in Switzerland, a country with a small but disproportionately high population of travelers. Therefore, we characterized all non-O157 STEC strains collected by the Swiss National Centre for Enteropathogenic Bacteria (Zurich, Switzerland) during 2000–2009, characterized these strains according to clinical and anamnestic data, and compared these results with data from other countries in Europe and the United States.

## Materials and Methods

### Strains

A total of 97 non-O157 STEC strains obtained from the Swiss National Centre for Enteropathogenic Bacteria were characterized. Strains were isolated during 2000–2009 from fecal samples of human patients with a reasonable clinical suspicion of infection with STEC. Samples sent to the Centre from hospitals or private practitioners are representative for Switzerland and the period screened.

### Serotyping

STEC isolates were serotyped by using O (O1–O186)–specific and H (H1–H56)–specific rabbit antiserum produced at the Federal Institute for Risk Assessment (Berlin, Germany). Nonmotile strains were investigated with respect to their flagellar genotypes by using PCR and *Hha*I digestion of PCR products as described (*3*).

### Strain Characterization

Fermentation of sorbitol was tested by using sorbitol MacConkey agar (Oxoid Ltd., Basingstoke, UK). PCRs targeting the *stx*1 and *stx*2 (*4**,**5*) *eae* (*6*), and *ehxA* (*7*) genes were performed as described.

### Genotyping

Pulsed-field gel electrophoresis (PFGE) was performed according to the PulseNet protocol (Centers for Disease Control and Prevention, Atlanta, GA, USA) (*8*) and by using restriction enzyme *Xba*I and the CHEF-DR III system (Bio-Rad, Hercules, CA, USA). Pulse times were ramped from 5 to 50 sec for 19 h at an angle of 120°. *Salmonella enterica* serovar Braenderup strain H9812 (BAA 664; American Type Culture Collection, Manassas, VA, USA) was used as a reference. GelCompar II software (Applied Maths NV, Sint-Martens-Latem, Belgium) was used for pattern comparison. PFGE patterns were considered clonally related if they had a similarity coefficient >80% (Dice similarity index and unweighted pair-group with arithmetic mean method).

### Antimicrobial Drug Susceptibility Testing

Strains were tested for antimicrobial drug resistance by the disk-diffusion method according to protocols of the Clinical and Laboratory Standards Institute (*9*). The panel of antimicrobial drug disks (Becton Dickinson, Sparks Glencoe, MD, USA) used contained ampicillin, amoxicillin/clavulanic acid, ceftazidime, cefalothin, ciprofloxacin, cefpodoxime, cefotaxime, cefuroxime, cefepime, cefoxitin, gentamicin, and tetracycline. *E. coli* strain 25922 (American Type Culture Collection) was used as a quality control.

## Results

### Strains

The 97 strains were isolated from 95 patients. Two STEC strains (968–03I and 968–03II; 2244–08I and 2244–08II) were isolated from 2 patients (Table A1). During the period of screening, an increasing number of strains per year were registered. This increase could have been caused by the official reporting system for STEC, which was initiated in Switzerland in 1999; or by a country-wide reporting program for HUS, which was initiated in Switzerland in 2004 and may have increased physician awareness for HUS.

### Anamnestic Data

**Figure 1:**
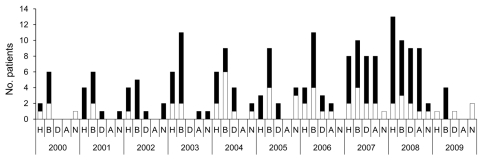
Anamnestic data for 97 non-O157 Shiga toxin–producing *Escherichia coli* (STEC) (black bar sections) and 44 O157 STEC (white bar sections) strains isolated from human patients, Switzerland, 2000–2009. H, hemolytic uremic syndrome; B, bloody diarrhea; D, nonbloody diarrhea; A, anemia; N, no anamnestic data available.

Anamnestic data for the 95 patients are shown in Figure 1. HUS developed in 38 patients (40%); 18 (47.4%) were male, 20 (52.6%) were female, and median age was <2 years (range <1–81 years). Bloody diarrhea was noted for 54 (56.8%) patients, nonbloody diarrhea for 22 (23.2%), and anemia for 16 (16.8%). For 7 patients, no anamnestic data were available. Ten (10.5%) patients were >60 years of age and 63 (66.3%) were <10 years of age. For some patients, there was epidemiologic evidence of contact with animals, traveling, or eating animal-derived food.

No data were available for use of antimicrobial drugs. However, it is generally assumed in Switzerland that no antimicrobial drugs should be given to patients with acute diarrhea if laboratory test results are not available.

### Characterization of STEC Serotypes and Virulence Genes

The strains belonged to 40 O:H serotype; 4 serotypes (O26:H11/H^–^; O103:H2, O121:H19, and O145:H28/H^–^) accounted for 46.4% of the strains (Table A1). No significant differences were found between most prevalent serogroups and symptoms observed.

When grown on sorbitol MacConkey agar, 80 (82.5%) strains fermented sorbitol and 17 (17.5%) did not. Forty-five (46.4%) strains had *stx*2 genes, 36 strains (37.1%) had *stx*1, and 16 (16.5%) strains had *stx*1 and *stx*2. Genes for enterohemolysin and intimin were detected in 75.3%, and 70.1% of the strains, respectively.

### PFGE Genotyping

**Figure 2:**
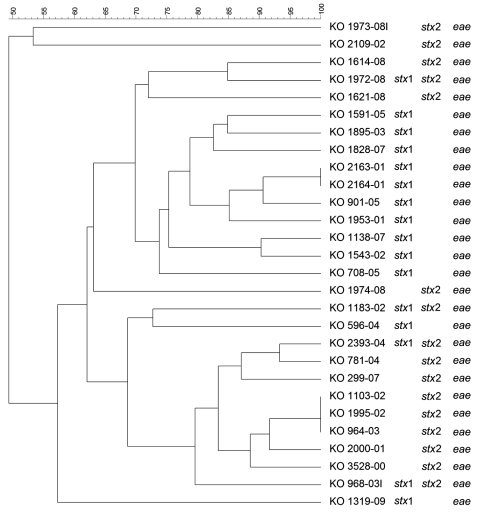
Dendrogram of Shiga toxin–producing *Escherichia coli* O26 strains isolated from human patients, Switzerland, 2000–2009. *stx*, Shiga toxin gene; *eae*, intimin gene. Scale bar indicates degree of similarity (%).

**Figure 3:**
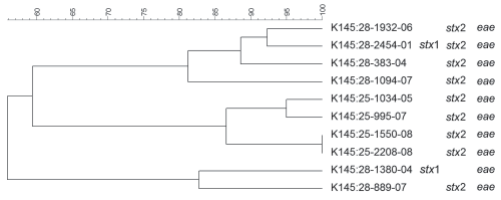
Dendrogram of Shiga toxin–producing *Escherichia coli* O145 strains isolated from human patients, Switzerland, 2000–2009. *stx*, Shiga toxin gene; *eae*, intimin gene. Scale bar indicates degree of similarity (%).

**Figure 4:**
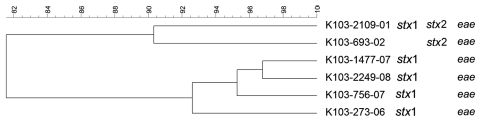
Dendrogram of Shiga toxin–producing *Escherichia coli* O103 strains isolated from human patients, Switzerland, 2000–2009. *stx*, Shiga toxin gene; *eae*, intimin gene. Scale bar indicates degree of similarity (%).

**Figure 5:**
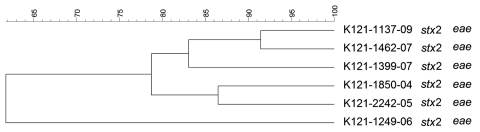
Dendrogram of Shiga toxin–producing *Escherichia coli* O121 strains isolated from human patients, Switzerland, 2000–2009 in Switzerland. *stx*, Shiga toxin gene; *eae*, intimin gene. Scale bar indicates degree of similarity (%).

PFGE conducted for strains of the most prevalent serogroups (O26, O103, O121, and O145) showed that the patterns of O26 strains were heterogeneous (similarity coefficient range 49%–94%) except for 5 strains (Figure 2). Patterns of O145 strains were also heterogeneous (similarity coefficient range 56%–95%) except for 2 strains (Figure 3). Patterns of O103 and O121 strains were heterogeneous (similarity coefficients ranges 86%–97% and 62%–91%, respectively) (Figures 4, 5). For O26 and O145 strains with the same PFGE patterns, no obvious epidemiologic link was observed between patients and different regions of Switzerland. Moreover, no information was available for about risk factors for these patients.

### Antimicrobial Drug Susceptibility Testing

All non-O157 STEC strains were susceptible to 5 antimicrobial drugs (ceftazidime, ciprofloxacin, cefotaxime, cefepime, and cefoxitin). Results are summarized in the online Table A1. Among 97 strains, 13 (13.4%) were resistant to ampicillin, 3 (3.1%) to amoxicillin/clavulanic acid, 12 (12.4%) to cefalothin, 1 (1%) to cefpodoxime, 1 (1%) to cefuroxime, 2 (2.1%) to gentamicin, and 21 (21.6%) to tetracycline. The most frequent combination was resistance to ampicillin, cefalothin, and tetracycline, which was detected in 8 isolates, once each with additional resistance to amoxicillin/clavulanic acid or gentamicin. One strain, 1972–08, appeared to hyperexpress a broad-spectrum β-lactamase (resistance to ampicillin, amoxicillin/clavulanic acid, cefalothin, cefuroxime, and cefpodoxime).

## Discussion

Over the 10-year study period (2000–2009), HUS caused by infection with non-O157 STEC was detected in 38 (40%) of the 95 patients investigated. This frequency of HUS cases in our study was higher than that in other studies (*10**,**11*). In contrast to other studies, in which all diarrhea samples were screened for STEC, the set of non-O157 strains in our study was isolated from patients with a reasonable clinical suspicion of infection with STEC. This fact could be the reason for a higher proportion of HUS and bloody diarrhea cases in our study group. Fourteen (36.8%) HUS cases were caused by STEC O26:H11; the remaining cases were caused by other serotypes. Of the patients with HUS, 20 (52.6%) were female and 18 (47.4%) were male. Furthermore, 30 (78.9%) were <5 years of age and only 4 (10.5%) were >60 years of age.

In a study in Minnesota, USA, over a 7-year period (2000–2006) in which 108 non-O157 STEC isolates were obtained, HUS did not develop in any of the patients; 57% were female (*10*). In Germany, Austria, and Australia, O111 strains were most frequently associated with HUS (*11*). In our study, 32 (84.2%) of the HUS isolates had *stx*2 as the only STEC gene (75%) or in combination with *stx*1 (25%), and 6 (15.8%) isolates had only *stx*1. This frequency of *stx2* in HUS isolates is similar to that in other countries, such as the United States (*12*).

Among the 30 detected STEC serogroups, O26 was most common (28 strains), followed by O145 (10 strains), O103, and O121 (6 strains each). These frequently found serogroups and others (O20, O113, O128, O146, O148, and O174) identified in our strain collection have also been found in sheep and cattle in Switzerland (*13**,**14*).

Our finding that O26 isolates (28.9%) were the most common non-O157 STEC serogroup found in Switzerland is similar to findings reported from Belgium, Germany, Japan, Spain, and the United States (*15**–**19*). Similar to other countries (*16*), in Switzerland, STEC O26:H11/H^–^ also caused several HUS cases.

Of the remaining major O groups, O145 (6 isolates of O145:[H28] and 4 isolates of O145:H25/H^–^) was the second most common non-O157 serogroup isolated in this study (10.3% of all isolates). Eight O145 isolates had positive results for *stx*2, *eae*, and *ehxA*; 1 isolate that did not ferment sorbitol had positive results for *stx*1, *eae*, and *ehxA*; and 1 isolate that had positive results for *stx*1 and *stx*2 also had positive results for *eae* and *ehxA*. These strain characteristics are similar to those of isolates from Finland and Germany (*20**,**21*). Four of the O145 strains were associated with bloody diarrhea, and 5 were associated with HUS, which is similar to symptom distribution associated with STEC O145 reported by Karch et al. (*18*).

The third most common non-O157 STEC serotypes isolated were O103:H2/H^–^ (6 strains) and O121:H19 (6 strains). In contrast to findings from Germany (*19*), none of the O103 strains from Switzerland were associated with HUS.

Among the STEC O121:H19 group, all but 1 of the strains were motile, fermented sorbitol, and were *stx*2, *eae*, and *ehxA* positive; the 1 exception was *ehxA* negative. Four patients had bloody diarrhea and HUS developed in 2 patients. These findings confirm the results of Johnson et al. (*11*), which showed that strains of serogroup O26, O103, O121, and O145 are more likely to be associated with cases of HUS.

Although STEC O111 is one of the most common serogroups in countries such as Germany, Austria, and Australia and is often associated with HUS (*11**,**22*), we found only 2 O111:H^–^ strains that had the H8 genotype. One of these strains had *stx*1, *eae*, *ehxA*, and the other strain had *stx*1, *stx*2, *eae* and *ehxA*. The patients from whom these 2 strains were isolated sought treatment for bloody diarrhea and HUS, respectively.

According to FoodNet 2009 (*23*), the most common non-O157 STEC serogroups in the United States are O26 (28.9%), O103 (20.0%), and O111 (14.9%). The frequency of O26 (28.9% of all isolates) in the United States is the same as that in Switzerland. However, the frequencies of STEC O103 and O111 in the United States are higher than those in Switzerland (STEC O103, 6.2%; STEC O111, 2.1%).

In Germany, STEC O91 (H14/H21) is currently the fourth most common STEC serogroup isolated (*24*). However, we have detected only 1 O91:H10, *stx*2-positive, *eae*- and *ehxA*-negative, sorbitol-fermenting strain in Switzerland during the past decade. This strain was isolated from a 60-year-old woman in whom HUS developed. STEC O91 isolates that express flagellar antigen H10 have been detected in different countries, albeit at low frequencies (*25**–**27*). O91 is usually *eae* negative and the most common serogroup isolated from adult patients (*25*). Another *eae*-negative serogroup is O117, which is often associated with travelers, mainly to Asia, Africa, and Cuba (*28*). We identified 3 (3.1%) O117:H7 strains that were *stx*1 positive and *stx*2, *eae*, and *ehxA* negative. This virulence pattern was identical to that for 20 STEC strains reported by Olesen et al. (*28*). One of our strains was associated with a patient who traveled to India, a country that has been reported as the origin of O117 infections (*29*). However, no information was available regarding travel for the other 2 patients in our study from whom the O117:H7 *stx*1-positive, *stx*2-, *eae*-, and *ehxA*-negative strains were isolated. Two of these patients had bloody diarrhea, but no clinical data were available for the third patient.

To detect genetic similarities and epidemiologic relationships among STEC strains, we performed PFGE on representatives of serogroups that occurred at high frequencies. O26 PFGE patterns were heterogeneous except for 2 strain sets, which had 2 and 3 strains without any obvious epidemiologic relationship to each other. Set 1 contained 2 *stx*1-positive, *eae*-positive strains isolated in 2001 (KO 2163–01 and KO 2164–01) from an 80-year-old woman and a 2-year-old-boy, respectively, each of whom had bloody diarrhea. Set 2 contained 3 *stx2*-positive, *eae*-positive strains isolated in 2002 and 2003 (KO 1103–02, KO 1995–02, and KO 964–03), from 3 female patients (1, 2, and 41 years of age), all of whom had HUS and bloody diarrhea. However, no information for risk factors, such as contact with animals, traveling, or eating animal derived-food, was available for these 5 patients.

PFGE patterns for O145 strains were also heterogeneous, except for 2 similar strains without any epidemiologic relationships to each other that were isolated in 2008 (K 145:25–1550–08 and K 145:25–2208–08; both *stx*2 and *eae* positive, 1 from a 2-year-old girl who was treated for HUS, and 1 from a 1-year-old boy who had bloody diarrhea). However, no information for risk factors, such as contact with animals, traveling, or eating animal-derived food, was available for these 2 patients. PFGE patterns of O103 and O121 strains were the most heterogeneous; none of the strains had the same patterns.

The *stx* gene distribution among all 97 non-O157 STEC isolates showed that 45 (46.4%) strains had only *stx*2, 36 (37.1%) had only *stx*1, and 16 (16.5%) had *stx*1 and *stx*2. Studies from Spain (*17*) and Finland (*20*) showed a similar distribution of *stx* genes among non-O157 STEC isolates. These findings are in contrast to those from the United States (Minnesota), in which non-O157 STEC strains generally showed a higher frequency of isolates that had *stx*1 (*10*).

The presence of the *eae* gene has been reported to have some predictive value for STEC seropathotypes that are associated with epidemic disease and consequently associated with severe disease such as bloody diarrhea and HUS (*12**,**30**,**31*). Therefore, we performed statistical analysis with multinomial regression and binary logistic regression for our data.

Strains associated with HUS, compared to those associated with bloody diarrhea, were more likely to harbor *stx*2 and *eae*, but the presence of only 1 of these virulence factors was not significantly associated. In our study, of 29 patients who provided *eae*-negative isolates, 17 had bloody diarrhea, 4 had bloody diarrhea and HUS, 4 had HUS, 1 had nonbloody diarrhea, and 3 had no clinical data available. Strains from the 8 patients with HUS comprised a variety of serogroups (O20, O82, O91, O148, O153, O181, and ONT); only 5 had the *stx*2 gene. In our study, 21.1% of isolates from HUS patients were *eae* negative. The 3 *eae*-negative, *stx*2-negative strains had only *stx*1 or *stx*1 and *ehxA*. Strains with such a pattern of virulence factors are notable because they are less likely to cause HUS than strains harboring *stx*2. However, these findings are consistent with epidemiologic data from other countries (*19**,**27*), which indicate that certain *eae*-negative STEC strains cause hemorrhagic diseases in humans. A report from the United States (Minnesota) indicated that non-O157 isolates that had only *stx*1 can cause severe illness (bloody diarrhea or HUS) (*32*).

Resistance to >1 of the 12 antimicrobial drugs tested was identified in 25 (25.8%) non-O157 STEC strains. This finding is consistent with results for a study in Spain, in which 238 (41%) of 581 non-O157 STEC strains were resistant to >1 of 26 antimicrobial drugs (*33*). In our study, 13 (13.4%) of 97 strains were resistant to ampicillin, 3 (3.1%) to amoxicillin/clavulanic acid, 12 (12.4%) to cefalothin, 1 (1%) to cefpodoxine, 1 (1%) to cefuroxime, 2 (2.1%) to gentamicin, and 21 (21.6%) to tetracycline. In STEC strains, the most frequent drug-resistance combination was resistance to ampicillin, cefalothin, and tetracycline, which was detected in 8 isolates, once with resistance to amoxicillin/clavulanic acid and once with resistance to gentamicin. Comparably, Schroeder et al. (*34*) tested 137 non-O157 *E*. *coli* human isolates (including 37 STEC strains) from the United States, Saudi Arabia, Argentina, Canada, Mexico, Zambia, and Singapore and reported STEC drug-resistance frequencies of 14% for ampicillin, 5% for amoxicillin/clavulanic acid, 11% for cefalothin, and 32% for tetracycline.

In conclusion, high genetic diversity within strains indicates that non-O157 Shiga toxin–producing *E. coli* infections in Switzerland most often occurred as single cases. Because little data are available for clinical non-O157 STEC infections in humans, our results may provide useful information for analysis of these strains.

## Appendix

**Table A1:** Serotypes and virulence factors of non-O157 STEC strains isolated from human patients, Switzerland, 2000–2009*

Strain	Serotype	Sorbitol fermentation	*stx*1	*stx*2	*eae*	*ehxA*	Disease	Drug resistance
645–01	O8:H16	+	+	+	–	+	BD	None
1655–05	O8:H30	+	+	–	+	+	BD	None
140–09	O20:[H30]	+	+	–	–	–	BD	AM, CF, TE
1402–02	O20:H30	+	+	–	–	–	HUS	AM, TE
1866–03	O20:H30	+	+	–	–	–	BD	AM, CF, TE
1591–05	O26: [H11]	+	+	–	+	+	D, HUS	None
1972–08	O26:[H11]	+	+	+	+	+	A, D, HUS	AM, AMC, CF, CPD, CXM
1974–08	O26:[H11]	+	–	+	+	+	A, D, HUS	TE
3528–00	O26:H11	+	–	+	+	+	HUS	None
1953–01	O26:H11	+	+	–	+	+	HUS	AMC, AM, CF, TE
2000–01	O26:H11	+	–	+	+	+	HUS	None
2163–01	O26:H11	+	+	–	+	+	BD	None
2164–01	O26:H11	+	+	–	+	+	BD	None
1103–02	O26:H11	+	–	+	+	+	HUS	None
1183–02	O26:H11	+	+	+	+	+	D, HUS	TE
1543–02	O26:H11	+	+	–	+	+	BD	None
1995–02	O26:H11	+	–	+	+	+	BD	AM, CF
2109–02	O26:H11	–	–	+	+	+	BD	None
964–03	O26:H11	+	–	+	+	+	BD, HUS	None
968–03 I	O26:H11	+	+	+	+	+	BD	TE
1895–03	O26:H11	+	+	–	+	+	BD	None
596–04	O26:H11	+	+	–	+	+	ND	None
781–04	O26:H11	+	-	+	+	+	D, HUS	None
2393–04	O26:H11	+	+	+	+	+	D, HUS	None
708–05	O26:H11	+	+	–	+	+	BD	None
901–05	O26:H11	+	+	–	+	+	BD	None
299–07	O26:H11	+	–	+	+	+	A, D, HUS	None
1138–07	O26:H11	+	+	–	+	+	BD	None
1828–07	O26:H11	+	+	–	+	+	BD	None
1614–08	O26:H11	+	–	+	+	+	BD, HUS	None
1621–08	O26:H11	+	–	+	+	+	A, D, HUS	None
1319–09	O26:H11	+	+	–	+	+	BD	TE
1973–08	O26:H34	+	–	+	+	–	D, HUS	None
1159–03	O55:H7	+	–	+	+	–	BD, HUS	None
1976–08	O55:H7	–	+	–	+	–	A, D, HUS	None
1384–03	O80:[H2]	+	–	+	+	+	BD	TE
1970–08	O80:[H2]	+	–	+	+	+	D, HUS	AM, CF, TE
2329–08	O82:H8	+	+	+	–	+	A, BD, HUS	None
1463–03	O91:H10	+	–	+	–	–	BD, HUS	None
878–03	O98:[H21]	+	+	–	+	+	BD	None
756–07	O103:[H2]	+	+	–	+	+	BD	None
2109–01	O103:H2	+	+	+	+	+	ND	None
693–02	O103:H2	+	–	+	+	+	ND	None
273–06	O103:H2	+	+	–	+	+	BD	None
1477–07	O103:H2	+	+	–	+	+	D	None
2249–08	O103:H2	+	+	–	+	+	BD	None
1805–06	O105:H18	–	+	+	–	+	BD	None
1086–00	O111: [H8]	+	+	–	+	+	BD	None
943–07	O111:[H8]	+	+	+	+	+	A, D, HUS	AM, CF, TE
2244–08 II	O113:H21	+	–	+	–	–	BD	None
968–03 II	O113:H4	+	+	+	–	+	BD	None
514–00	O117:H7	–	+	–	–	–	BD	TE
1117–00	O117:H7	–	+	–	–	–	BD	None
883–02	O117:H7	–	+	–	–	–	ND	None
1850–04	O121:H19	+	–	+	+	+	HUS	None
2242–05	O121:H19	+	–	+	+	+	BD	None
1249–06	O121:H19	+	–	+	+	+	BD	None
1399–07	O121:H19	+	–	+	+	+	BD	None
1462–07	O121:H19	+	–	+	+	+	A, D, HUS	None
1137–09	O121:H19	+	–	+	+	–	BD	None
130–03	O123:H2	+	+	–	+	+	BD	None
2384–04	O128:H2	+	–	+	–	+	BD	AM, CF
568–09	O128:H45	–	–	+	–	–	BD	TE
1904–06	O128ac:H2	+	+	+	–	–	BD	None
2440–08	O144:[H25]	–	–	+	+	+	BD	None
1550–08	O145:[H25]	+	–	+	+	+	A, HUS	None
2454–01	O145:[H28]	+	+	+	+	+	D, HUS	None
383–04	O145:[H28]	+	–	+	+	+	BD	None
1380–04	O145:[H28]	–	+	–	+	+	BD	None
1932–06	O145:[H28]	+	–	+	+	+	D, HUS	None
889–07	O145:[H28]	+	–	+	+	+	A, D, HUS	TE
1094–07	O145:[H28]	+	–	+	+	+	A, BD	None
1034–05	O145:H25	+	–	+	+	+	ND	None
995–07	O145:H25	+	–	+	+	+	A, D, HUS	TE
2208–08	O145:H25	+	–	+	+	+	BD	None
001–06	O146:H28	+	–	+	–	–	BD	None
128–03	O148:H8	+	–	+	–	–	A, HUS	AM, CF, TE
1146–05	O153:[H4]	+	+	–	–	+	HUS	None
1586–08	O153:[H40]	+	+	+	–	–	A, BD, D, HUS	AM, CF, GM, TE
2244–08 I	O156:H25	–	+	–	+	+	BD	None
1933–06	O167:H32	+	+	–	–	–	BD	None
1533–01	O174:H2	+	+	+	–	+	BD	AMC, AM, CF, GM
2436–08	O174:H21	+	–	+	–	–	ND	TE
908–02	O177:[H25]	–	–	+	+	+	BD	None
1237–04	O181:H49	+	+	+	–	+	HUS	None
1093–00	O183:[H18]	–	+	+	–	+	BD	None
1205–07	ONT:H30	+	+	–	–	–	BD, HUS	AM, CF, TE
2537–03	ONT:H45	–	–	+	–	–	ND	None
722–04	Or:–	+	+	–	+	+	D	None
1677–05	Or:–	+	+	–	+	+	BD	None
2841–01	Or:[H25]	–	–	+	+	+	HUS	None
1078–06	Or:[H25]	–	–	+	+	+	HUS	None
2349–08	Or:[H25]	+	–	+	+	+	A, HUS	None
2103–05	Or:H25	+	–	+	+	+	D, HUS	None
1325–02	Or:H31	+	–	+	–	–	BD	None
012–06	Or:H7	–	+	–	–	–	BD	TE
1202–06	Or:H7	–	+	–	–	–	D	TE

*STEC, Shiga toxin–producing *Escherichia coli*; *stx*, Shiga toxin; *eae*, intimin; *ehxA*, enterohemolysin; +, positive; –, negative; BD, bloody diarrhea; [ ], nonmotile strains investigated with respect to their flagellar genotypes by PCR; AM, ampicillin; CF, cephalothin; TE, tetracycline; HUS, hemolytic uremic syndrome; D, nonbloody diarrhea; A, anemia; AMC, amoxicillin/clavulanic acid; CPD, cefpodoxime; CXM, cefuroxime; ND, no data; GM, gentamicin; ONT, nontypeable; Or, O rough.
